# Experimental and numerical optimization of pressurized air vessel performance for water hammer mitigation

**DOI:** 10.1038/s41598-025-23962-4

**Published:** 2025-11-12

**Authors:** A. M. Hamed, A. M. Abdulaziz

**Affiliations:** Department of Mechanical Power Engineering, Faculty of Engineering, Ain Shams University, Cairo, 11517 Egypt

**Keywords:** Water hammer, Transient flow, Pressurized air vessel, Mathematical modelling, Throttling, Orifice diameter, Water volume fraction ratio, Experimental study, Energy science and technology, Engineering, Physics

## Abstract

Controlling water hammer pressure is essential, necessitates a transient surge analysis to identify critical pressure points along a pipeline system. A pressurized air vessel is a pressure control device used to control both positive and negative pressure fluctuations. This study investigates three key parameters that affect the sizing of the pressurized air vessel: orifice diameter (the throttling aperture), the vessel diameter, and water volume fraction ratio. A mathematical model, developed using the FORTRAN programming language and based on the unsteady one-dimensional momentum and continuity equations, determines the optimal sizing of these parameters. These equations are solved using the method of characteristics, and the pressurized air vessel is mathematically modelled as a quasi-one-dimensional flow system. An experimental test rig, equipped with a rapid closing solenoid valve and pressure sensors, is used to validate the mathematical model results. Both the experimental and numerical results demonstrate the effectiveness of the pressurized air vessel to dampen water hammer pressure. The findings indicate that the throttling action has a significant effect on the required size of the pressurized air vessel. This study presents a novel approach that provides quantitative insights into key parameters that affect the performance of the pressurized air vessel by using the combined modelling and experimental validation. The orifice diameter is the most influential parameter on the water hammer head, vessel air head, and water level inside the vessel.

## Introduction

Water hammer is a pressure surge caused by a sudden change in flow velocity in the pipeline. This phenomenon is often accompanied by a loud banging noise, which is why it is referred to as water hammer. The transient pressure waves can force a system to operate under conditions for which it was not designed^1,2^. Water hammer is a significant hazard for water pipelines, as the resulting pressure build-up can exceed the pressure rating of the pipes. Therefore, the use of a transient control device has become indispensable for the liquid piping line. Instant valve closure is one of the causes of water hammer, as it results in abrupt change in the head above the predetermined rated values^3^. Various devices and methods are used to control transient pressure, including pressurized air vessels^4^, surge tanks^5^, pressure relief valves^6,7^, and polymeric pipe sections near the transient source^8–10^. A correctly designed pressurized air vessel can provide the necessary safety precautions to resist transient pressure, such as that caused by a sudden power cutoff in a pumping system^11^. Therefore, an accurate flow simulation model and an iterative optimization process, incorporating unsteady-state friction, pump rotational inertial, valve closing time, head losses along connecting pipes, and wave celerity, are necessary to design a viable pressurized air vessel^1,4,12,13^.

In general, the performance of a pressurized air vessel depends on many parameters, such as its size, initial gas volume, thermodynamic behavior of the gas, the diameter of pipes that connect the pressurized air vessel to the main pipeline, the Reynolds number at the steady-state flow, the orifice discharge coefficient and the installation location, among others^14–16^. In addition, previous studies have indicated that a large, pressurized air vessel with a greater initial gas volume imparts superior water hammer protection^13,16^. According to Sattar et al.^17^, the minimum initial air volume should be in the range of 20% to75% of the total tank volume. Noting that, a larger pressurized air vessel with a higher volume is not cost-effective. Some researchers have studied the pressure vessel transient pressure protection performance using various connecting pipe configurations^14,18^. They further revealed that it is possible to reduce the required size of the vessel by appropriately adjusting the connecting pipe’s diameter and inflow/outflow losses^17,19^.

Water pipeline networks involve both substantial capital and operating costs, which have increased in recent decades due to rising component prices. To maintain system safety, transient control devices are essential to prevent pressures from exceeding design limits^20^. Surge analysis is therefore performed to predict unfavorable flow scenarios and identify suitable control measures^21^. The Joukowsky equation and one-dimensional governing equations based on mass and momentum conservation are commonly used to model transient flow, with the method of characteristics providing an efficient solution approach^3,22,23^. Among various water hammer control methods, pressurized air vessels have been widely studied. A pressurized air vessel, containing liquid at the base and compressed air above, utilizes air compressibility to attenuate pressure surges^24,25^. It offers advantages over many other devices by effectively managing both positive and negative pressure transients^26^, with performance influenced by factors such as vessel size, entrapped air volume, and pipeline profile^27–31^. Since water hammer occurs over short intervals, the trapped air process can be treated as adiabatic with a polytropic index of 1.2^28,29^. Design charts, experimental investigations, and optimization studies—such as those using Genetic Algorithms—have confirmed the effectiveness of air vessels in mitigating water hammer, regardless of installation orientation^28–36^.

The literature survey revealed that the effect of orifice diameter, vessel volume, and water volume fraction (WVFR) ratio still need further investigation. The current study presents a novel approach that provides quantitative insights into these key parameters that affect the performance of the pressurized air vessel by using the combined modelling and experimental validation. The main objective of the current study is to optimize the above mentioned three parameters to dampen the water hammer phenomenon. This was carried experimentally and theoretically. A mathematical model, created using FORTRAN programming language, was established. The obtained results were validated against the experiments.

## Methodology

The transient flow is governed by two fundamental partial differential equations: conservation of mass and conservation of momentum^11^. Since the pipe lengths are long relative to their diameters, the flow is simplified to one-dimensional unsteady flow. The test system consists of a long pipe with a pump at one end and a rapid closing valve (RCV) at the other end. The pressurized air vessel is installed just before the valve to dampen the water hammer effect. In the current study, the orifice diameter is in the range of 1 to 6 mm, while the water volume fraction ratio (WVFR) is in the range of 53% to 78% of the total vessel volume.

### Governing equations

Conservation of momentum^37^

1 $$\frac{\partial V}{{\partial t}} + \frac{1}{\rho }\frac{\partial p}{{\partial x}} + g\frac{dz}{{dx}} + \frac{f}{2D}V\left\lceil V \right\rceil = 0$$

where *V* is the flow velocity *p* is the fluid pressure, *z* is the elevation, *t* is time, *x* is the distance along pipe, $\rho$ is the fluid density,* g* is the gravitational acceleration, *D* is the pipe diameter and *f* is the friction factor.

Conservation of mass^37^

2 $$a^{2} \frac{\partial V}{{\partial x}} + \frac{1}{\rho }\frac{\partial p}{{\partial t}} = 0$$

where *a* is the speed of sound of water in pipes ^37^

3 $$a = \frac{{\sqrt {K/\rho } }}{{\sqrt {1 + \frac{K D}{{E e}}} }}$$

where *K* is Bulk modulus of water, *E* is Young’s modulus of the pipe material and *e* is the thickness of the pipe.

**Fig. 1:**
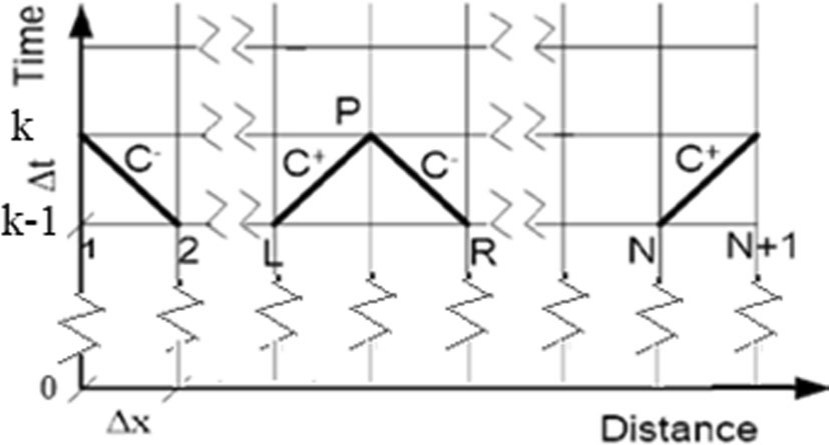
One-dimensional computation grid of the physical problem.

The method of characteristics is used to transform the partial differential equations (PDEs) (1) and (2) to ordinary differential equations (ODEs) valid along certain lines called the characteristic lines, $C^{ + }$ and $C^{ - }$. These ordinary differential equations (ODEs) are then solved using finite difference explicit scheme to get both the velocity and head at each mesh point along the pipe. Since we are interested in the fluid head and velocity, the substitution of *p* with (*H-z*) is used. The computational grid used in this mathematical modelling is shown in Fig. 1 where $\Delta x$ is the mesh distance and $\Delta t$ is the time step. For an interior point, the characteristic equations are

4 $$C^{ + } :(V_{p}^{\left( k \right)} - V_{L}^{{\left( {k - 1} \right)}} ) + \frac{g}{a}\left( {H_{p}^{\left( k \right)} - H_{L}^{{\left( {k - 1} \right)}} } \right) + \frac{f}{2D} \times \Delta t \times V_{L}^{{\left( {k - 1} \right)}} \times \left| {V_{L}^{{\left( {k - 1} \right)}} } \right| = 0$$

5 $$C^{ - } :(V_{p}^{\left( k \right)} - V_{R}^{{\left( {k - 1} \right)}} ) - \frac{g}{a}\left( {H_{p}^{\left( k \right)} - H_{R}^{{\left( {k - 1} \right)}} } \right) + \frac{f}{2D} \times \Delta t \times V_{R}^{{\left( {k - 1} \right)}} \times \left| {V_{R}^{{\left( {k - 1} \right)}} } \right| = 0$$

where *H* is the fluid head, suffix *p* denotes the point under consideration, *L* is the left value, *R* is the right value, *k* is the new value in time, and k−1 is the previous value in time.

### Boundary conditions

The accuracy of the simulation results depends on defining the boundary conditions properly. Proper boundary conditions guarantee conservation of mass and momentum across system boundaries. Without these boundary conditions, the solution of the governing equations of the mathematical model could yield non-physical results, such as fluid entering/exiting incorrectly.

- Constant speed pump at pipe entrance

The boundary condition at pipe inlet is defined by the Negative Characteristic equation (${C}^{-}$) and the pump characteristic pump equation. Applying Eq. (4) between the first and second mesh yield one equation at left boundary. Referring to Fig. 1:

6 $$C^{ - } :(V_{1}^{\left( k \right)} - V_{2}^{{\left( {k - 1} \right)}} ) - \frac{g}{a}\left( {H_{1}^{\left( k \right)} - H_{2}^{{\left( {k - 1} \right)}} } \right) + \frac{f}{2D} \times \Delta t \times V_{2}^{{\left( {k - 1} \right)}} \times \left| {V_{2}^{{\left( {k - 1} \right)}} } \right| = 0{ }$$

where k means the new property in time domain while k−1 means the previous time step.

The second boundary equation comes from the pump equation.

7 $$H_{1}^{k} = c_{1} \left( {V_{1}^{k} } \right)^{2} + c_{2} V_{1}^{k} + c_{3}$$

where *c*_*1*_, *c*_*2*_ and *c*_*3*_ were obtained from the experimental performance of the pump.

Solving Eqs. (6) and (7), the head, $H_{1}^{k}$ and velocity, $V_{1}^{k}$ at the pipe’s left end can be obtained.

(b) Rapid closure valve (RCV) at pipe far end

The boundary condition at pipe outlet is defined by the positive characteristic equation $(C^{ + } )$ and the valve closure equation. The Positive Characteristic equation at pipe end (Eq. 5) is applied between mesh N and N + 1. Referring to Fig. 1:

8 $${\text{C}}^{ + } :({\text{V}}_{{{\text{N}} + 1}}^{{\left( {\text{k}} \right)}} - {\text{V}}_{{\text{N}}}^{{\left( {{\text{k}} - 1} \right)}} ) + \frac{{\text{g}}}{{\text{a}}}\left( {{\text{H}}_{{{\text{N}} + 1}}^{{\left( {\text{k}} \right)}} - {\text{H}}_{{\text{N}}}^{{\left( {{\text{k}} - 1} \right)}} } \right) + \frac{{\text{f}}}{{2{\text{D}}}} \times \Delta {\text{t}} \times {\text{V}}_{{\text{N}}}^{{\left( {{\text{k}} - 1} \right)}} \times \left| {{\text{V}}_{{\text{N}}}^{{\left( {{\text{k}} - 1} \right)}} } \right| = 0$$

The Rapid Closure Valve (RCV) closes according to the following linear equation:

9a $${\text{V}}_{{{\text{N}} + 1}}^{{\left( {\text{k}} \right)}} = {\text{V}}_{0} \left( {1 - \frac{{\text{t}}}{{{\text{T}}_{{\text{c}}} }}} \right),0 \le {\text{t}} \le {\text{T}}_{{\text{c}}}$$

where V_o_ is the initial fluid velocity at valve entrance just before valve start closing, T_c_ is the valve closing time (from fully open to fully close). After the valve is fully closed the fluid velocity at pipe end is zero.

9b $${\text{V}}_{{{\text{N}} + 1}}^{{\left( {\text{k}} \right)}} = 0,{\text{t}} > {\text{T}}_{{\text{c}}}$$

Solving Eqs. (8) and (9), both head,${\text{ H}}_{{{\text{N}} + 1}}^{{\left( {\text{k}} \right)}}$ and velocity, ${\text{V}}_{{{\text{N}} + 1}}^{{\left( {\text{k}} \right)}} ,$ at pipe far end can be calculated.

(c) Pressurized air vessel

**Fig. 2:**
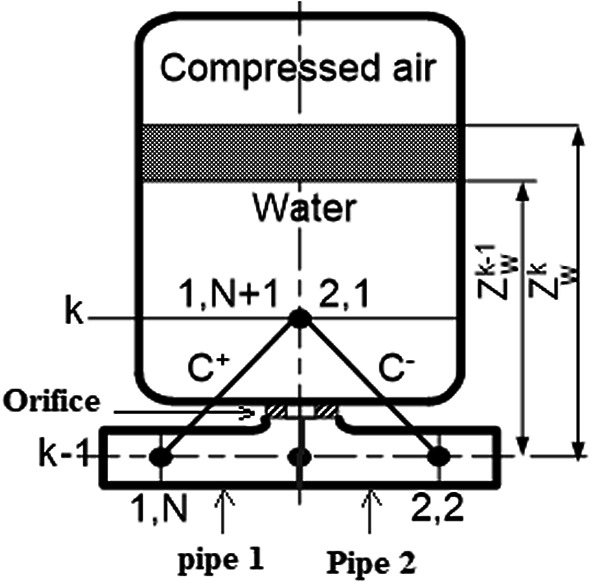
Pressurized air vessel model.

The computational mesh for the pressurized air vessel is clearly shown in Fig. 2. The pipe connected to the pressurized air vessel is composed of two pipes (pipe 1 and pipe 2), one on the left and the other is on the right. Both pipes are connected to the vessel through an orifice fitted in a short pipe. The whole group is considered a T-junction of constant pressure. The set of governing equations^11^ are explained as follows,

1. The positive and negative characteristics for left and right pipes are:
  10 $${\text{C}}^{ + } :({\text{V}}_{{1,,{\text{N}} + 1}}^{{\left( {\text{k}} \right)}} - {\text{V}}_{{1,{\text{N}}}}^{{\left( {{\text{k}} - 1} \right)}} ) + \frac{{\text{g}}}{{\text{a}}}\left( {{\text{H}}_{{1,{\text{N}} + 1}}^{{\left( {\text{k}} \right)}} - {\text{H}}_{{1,{\text{N}}}}^{{\left( {{\text{k}} - 1} \right)}} } \right) + \frac{{\text{f}}}{{2{\text{D}}_{1} }} \times \Delta {\text{t}} \times {\text{V}}_{{1,{\text{N}}}}^{{\left( {{\text{k}} - 1} \right)}} \times \left| {{\text{V}}_{{1,{\text{N}}}}^{{\left( {{\text{k}} - 1} \right)}} } \right| = 0$$
  11 $${\text{C}}^{ - } :({\text{V}}_{2,1}^{{\left( {\text{k}} \right)}} - {\text{V}}_{2,2}^{{\left( {{\text{k}} - 1} \right)}} ) - \frac{{\text{g}}}{{\text{a}}}\left( {{\text{H}}_{2,1}^{{\left( {\text{k}} \right)}} - {\text{H}}_{2,2}^{{\left( {{\text{k}} - 1} \right)}} } \right) + \frac{{\text{f}}}{{2{\text{D}}_{2} }} \times \Delta {\text{t}} \times {\text{V}}_{2,2}^{{\left( {{\text{k}} - 1} \right)}} \times \left| {{\text{V}}_{2,2}^{{\left( {{\text{k}} - 1} \right)}} } \right| = 0$$
  The suffix of any variable has two numbers. The first is either 1 or two which means either is pipe 1 or pipe 2. The second number is either 1 or N + 1 which means pipe entrance or pipe outlet. ${\text{V}}_{1,\text{N}+1}^{\left(\text{k}\right)}$ and ${\text{H}}_{1,\text{N}+1}^{\left(\text{k}\right)}$ denote the velocity and head at the end of the pipe 1 (left) at new time step. ${\text{V}}_{\text{2,1}}^{(\text{k})}\text{ and }{\text{H}}_{\text{2,1}}^{(\text{k})}$ denote the velocity and head at the entrance of pipe 2 (right) at new time step. k−1 denotes the old-time step.
2. A constant pressure model is assumed at the junction node (new time step).
  12 $${\text{H}}_{{1,{\text{N}} + 1}}^{{\text{k}}} = {\text{H}}_{2,1}^{{\text{k}}}$$
3. Continuity equation across the junction (at new time step)
  13 $${\text{V}}_{{1,{\text{N}} + 1}}^{{\left( {\text{k}} \right)}} \times {\text{A}}_{1} - {\text{V}}_{2,1}^{{\left( {\text{k}} \right)}} \times {\text{A}}_{2} = {\text{Q}}_{{{\text{p}}_{{{\text{orifice}}}} }}^{{\left( {\text{k}} \right)}}$$
  where A is the pipe cross area, p is the junction point and Q is the volume flow rate.
4. The new water level in the vessel is
  14 $${\text{Z}}_{{\text{w}}}^{{\text{k}}} = {\text{Z}}_{{\text{w}}}^{{{\text{k}} - 1}} + \frac{{\left( {{\text{Q}}_{{{\text{p}}_{{{\text{orifice}}}} }}^{{\left( {\text{k}} \right)}} + {\text{Q}}_{{{\text{p}}_{{{\text{orifice}}}} }}^{{\left( {{\text{k}} - 1} \right)}} } \right)\Delta {\text{t}}}}{{2{\text{A}}_{{\text{v}}} }}$$
  where Z is the water height in the vessel, suffix w means water and suffix v means valve.
5. The new trapped air volume in the vessel is
  15 $$\forall_{{{\text{air}}}}^{{\text{k}}} = \forall_{{{\text{vessel}}}} - {\text{A}}_{{{\text{vessel}}}} \times {\text{Z}}_{{\text{w}}}^{{\text{k}}}$$
  where $\forall$ means volume.
6. The trapped air located on the top level of the tank undergoes an isentropic compression
  16 $${\text{p}}_{{{\text{air}}}}^{{\text{k}}} = {\text{ p}}_{{{\text{air}}}}^{{{\text{k}} - 1}} \left( {\frac{{\forall_{{{\text{air}}}}^{{{\text{k}} - 1}} }}{{\forall_{{{\text{air}}}}^{{\text{k}}} }}} \right)^{{\upgamma }}$$
  where ${\upgamma }$ is the polytropic exponent.
7. The new head at junction point is
  17 $${\text{H}}_{{1,{\text{N}} + 1}}^{{\text{k}}} = {\text{H}}_{2,1}^{{\text{k}}} = {\text{Z}}_{{\text{w}}}^{{\text{k}}} + \frac{{({\text{p}}_{{{\text{air}} - 10^{5} )}}^{{\text{k}}} }}{9810} + \frac{{{\text{Q}}_{{{\text{p}}_{{{\text{orifice}}}} }}^{{2\left( {\text{k}} \right)}} \times \left( {1 - {\upbeta }^{4} } \right)}}{{2{\text{C}}_{{\text{d}}}^{2} \times {\text{A}}_{{{\text{orifice}}}}^{2} \times 9.81}}$$
  where $\upbeta$ is the orifice diameter divided by the pipe diameter.

There are eight unknowns in eight Eqs. (10)–(17), which can be solved by trial and error. A computer simulation program is written in FORTRAN to solve Eqs. (4) and (5) for the interior pipe points, Eqs. (6) and (7) for the pipe entrance, Eqs. (8) and (9) at the pipe end, and Eqs. (10)–(17) for the pressurized air vessel. The model results are compared with the experimental results as will be explained later.

## Experimental test rig setup

**Fig. 3:**
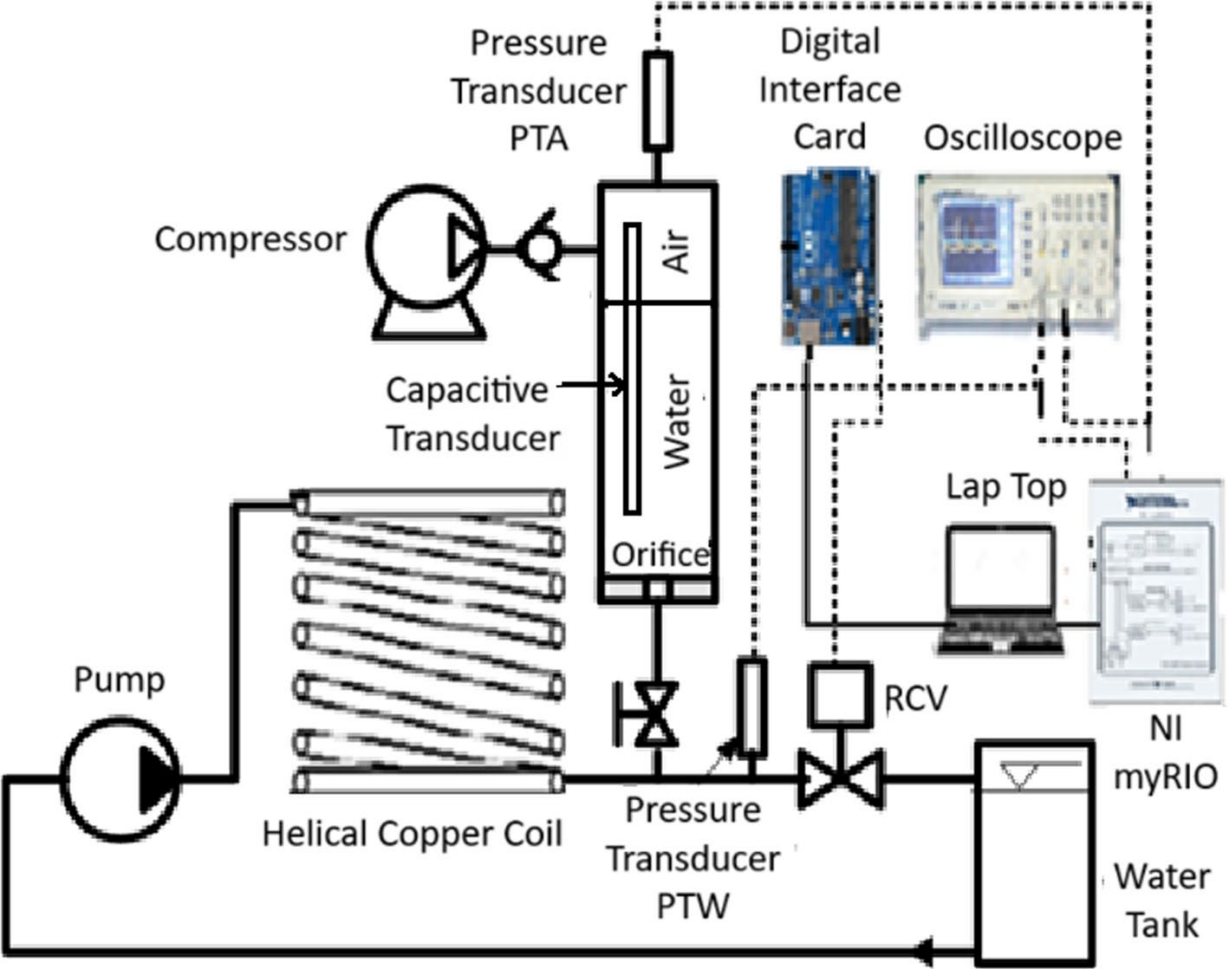
Experimental test rig set-up.

The experimental test rig, shown in Fig. 3, is composed of a closed water loop connected to a water tank at a constant head, a centrifugal pump, and a copper coil that ends with a rapid closing magnetic valve (RCV). The coil dimensions are 15 m long and 11.5 mm internal diameter. A pressurized air vessel of height 55 cm and diameter 5 cm is installed upstream the rapid closing magnetic valve (RCV) and is fabricated from acrylic transparent glass to facilitate the monitoring of the water level inside it. To maintain the volume of air at a pre-set value; the pressurized air vessel is connected to a constant pressure compressed air source to supply it with the required quantity of air. A pressure transducer, PTW, is mounted very close to the RCV to measure the water pressure during water hammer. This transducer’s measuring range is − 1 to 40 bar and has an accuracy 0.5% of full scale. Another pressure transducer, PTA, is mounted on the top of the pressurized air vessel to measure the air pressure inside the air vessel. Both pressure transducers are calibrated against pre-calibrated Bourdon tube pressure gauges. The water level inside the pressurized air vessel is measured by using a capacitive transducer, as shown in Fig. 3.

The rapid closing valve (RCV) has a maximum closing time of 20 ms. The RCV solenoid receives a signal from a control circuit to open for a predetermined time and close very rapidly. The pressure signals are monitored on a digital oscilloscope and recorded using NI myRIO data acquisition card which has 16 bits resolution and maximum sampling rate of 200 k-sample/s.

The correct choice of RCV should be based on the well-known water hammer relation:

$${\text{T}}_{{\text{c}}} < \frac{{2{\text{L}}}}{{\text{a}}},\;{\text{T}}_{{\text{c}}} < \frac{2 \times 15}{{1000}},\;{\text{T}}_{{\text{c}}} < 30{\text{ ms }}$$

where ${\text{T}}_{\text{c}}$ is the closing time of RCV, L is the pipe length and a is the sonic speed of water in the pipe coil. If the closing time is not less than 30 ms, the water hammer will not occur. The closing time of RCV is 20 ms which fulfils the above requirements. The pressurized air vessel, shown in Fig. 3, has an orifice hole located at the connection between the pressurized air vessel and pipeline T junction. Initially, the experiment is operated while isolated from the pressurized air vessel by closing the ball valve installed between the main pipe and the pressurized air vessel. The RCV is operated to create a water hammer, and the pressure rise is recorded via pressure transducer [PTW]. Thereafter, the test is repeated with connecting the pressurized air vessel where the water enters the pressurized air vessel to a certain level based on force balance.

## Results and discussion

### Validation of water hammer head without pressurized air vessel

**Fig. 4:**
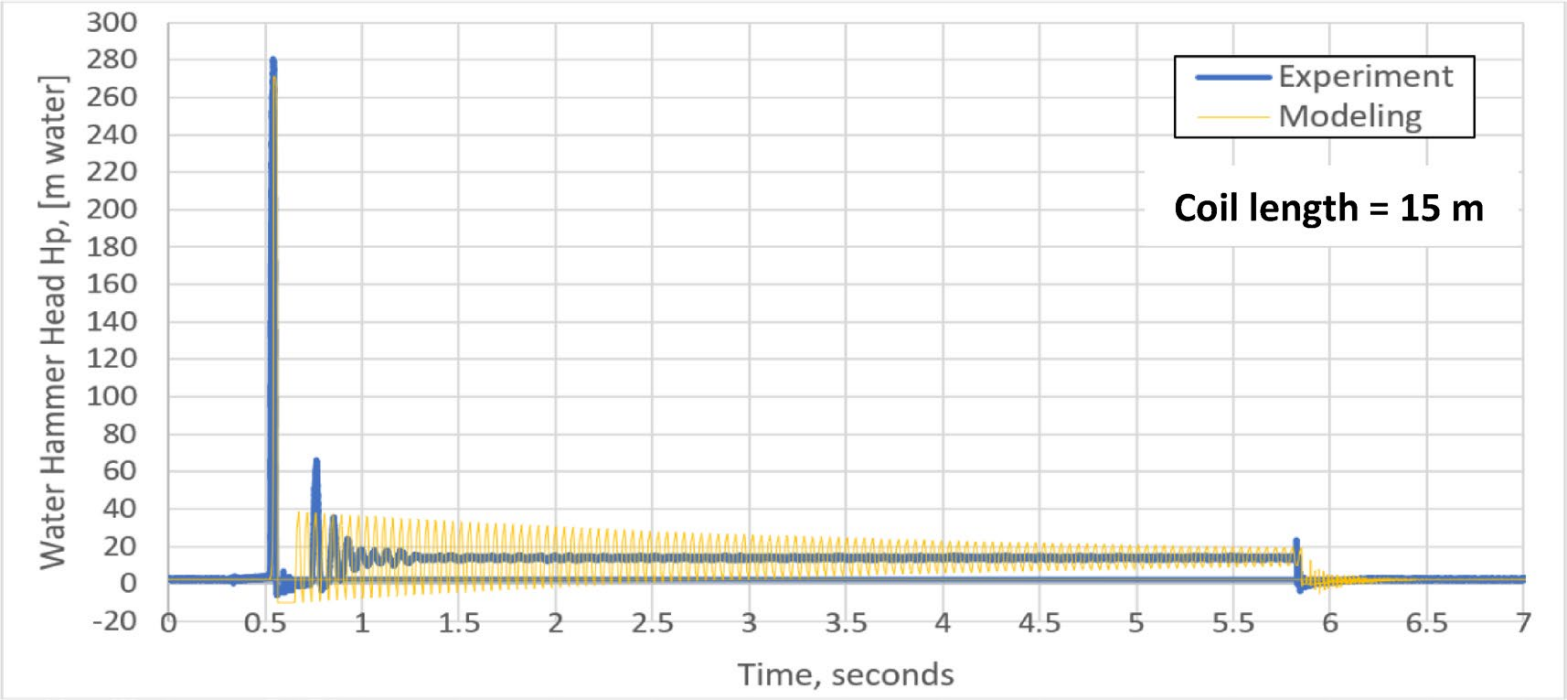
Experimental and theoretical water hammer head for a pipe coil of 15 m length without pressurized air vessel.

**Fig. 5:**
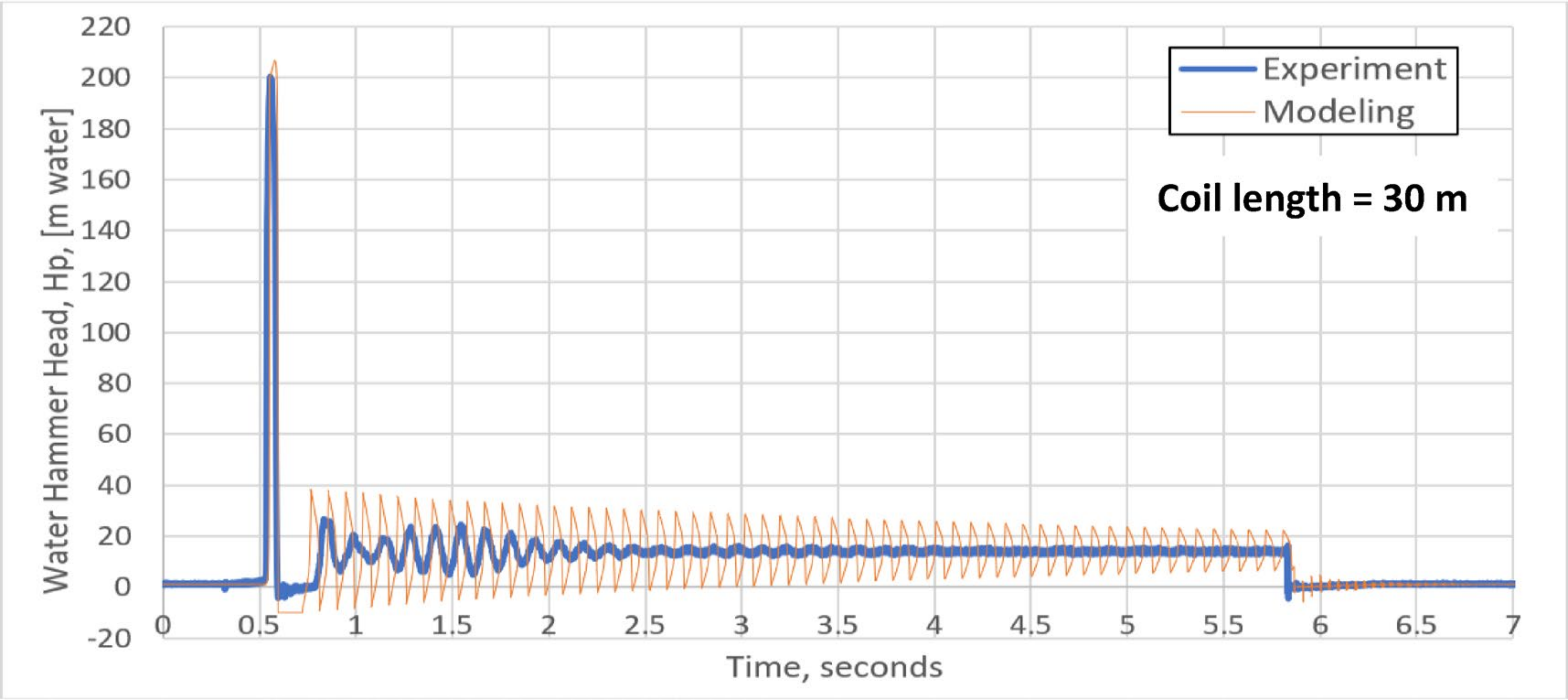
Experimental and theoretical water hammer head for a pipe coil of 30 m length without pressurized air vessel.

The water hammer standard test is applied by running the pump until steady flow of water is reached through the pipe coil. The rapid closing valve closes suddenly at 0.5 s and is kept closed for 5.2 s, after that it opens. Figures 4 and 5 show the variation of water hammer head H_P_ with time (without pressurized air vessel) for pipe coils of 15 m and 30 m length respectively. Due to sudden valve closure, the kinetic energy of the flowing water is converted to a pressure head rise $\Delta H$ according to the well-known formula^37^:

18 $$\Delta H = \frac{a \Delta V}{g}$$

where a is the sonic speed, $\Delta V$ is the steady flow velocity and g is the acceleration gravity.

As soon as the valve closes, a shoot-up in the water hammer head appears. The pressure falls suddenly because the inertia of the moving water keeps it flowing after the initial compression, causing a local “stretching” and reduction of pressure. Reflections at system boundaries reinforce this effect. The pressure can suddenly fall below steady-state pressure. This explains the sharp oscillation of pressure during water hammer (rise → fall → rise, etc.). Finally, the pressure stabilizes after the water hammer because the oscillating pressure waves gradually lose energy through friction, damping, and other dissipative mechanisms, until the fluid reaches a new steady-state equilibrium.

Both the experimental and the simulation results for pipe coil length of 15 and 30 m, are presented in Figs. 4 and 5, respectively. There is a good agreement between both these results. Due to the water hammer phenomenon, the maximum experimental and simulated water hammer heads for a pipe coil length of 15 m are 280.7 m water and 271.1 m water, respectively, corresponding to a deviation of 3.42% in the simulated results as shown in Fig. 4. These values are in a good agreement with the water hammer relation (a Δv/g = 1000 × 2.78 / 9.8 = 283.38 m water). Negative pressure values show up in the early stage of water hammer. In addition, there is a second sharp water hammer head of 63.37 m water for the experiment and 37.71 m water for the model. The periodical time of the pressure wave is nearly 0.0455 s (Fig. 4) which is very close to the well-known water hammer relation (4L /a).

**Fig. 6:**
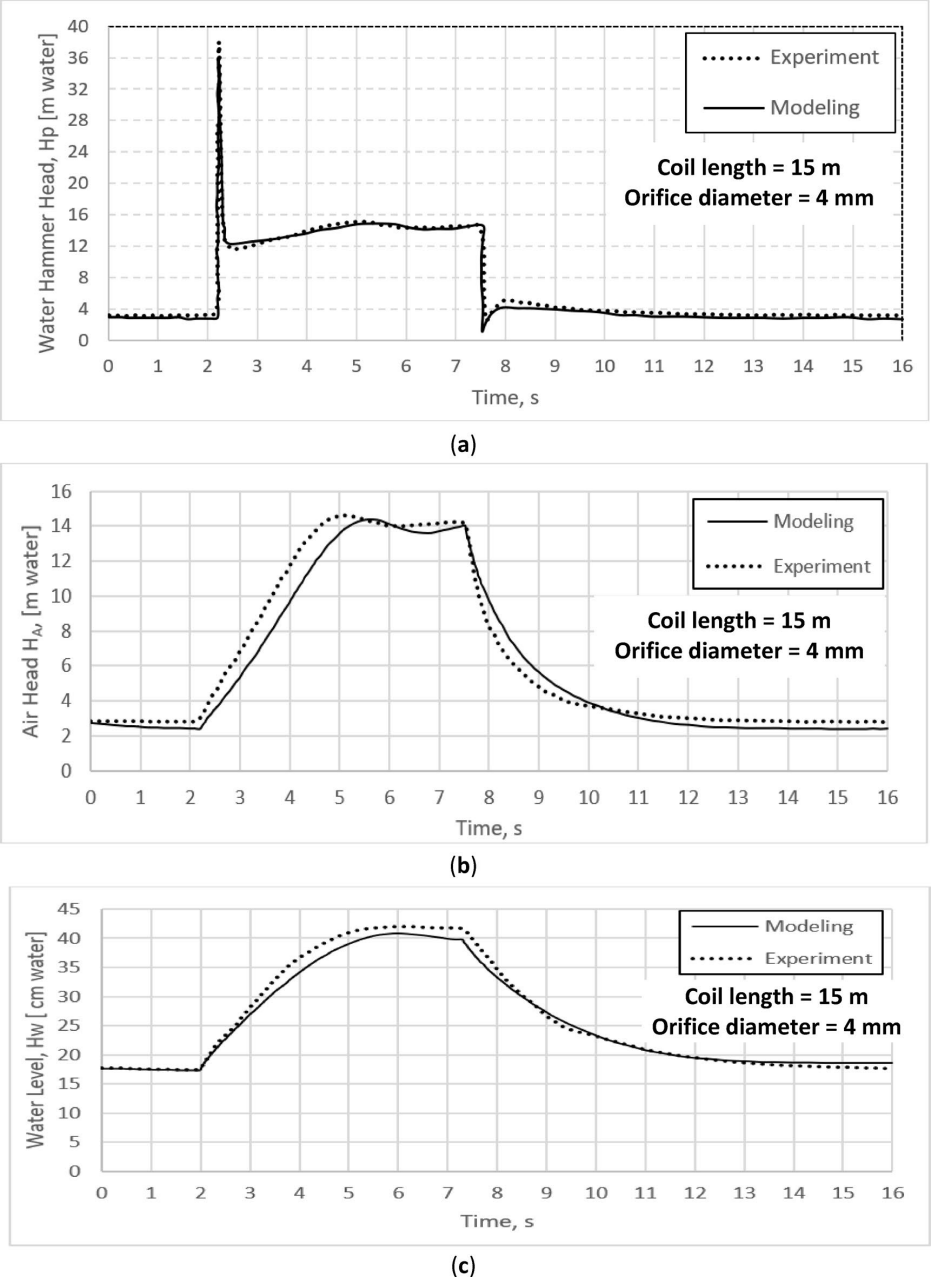
(**a**) Validation of the instantaneous water hammer head with time with pressurized air vessel**.** (**b**) Validation of the instantaneous air head with time with pressurized air vessel. (**c**) Validation of the instantaneous water level with time with pressurized air vessel.

Increasing the pipe coil length to 30 m will lead to a decrease in both the maximum experimental and simulated water hammer head to 201.25 m water and 203.99 m water, respectively with a deviation of 1.34% between them, as shown in Fig. 6. This reduction in the pressure rise is due to the reduction of the flow velocity due to the increase in friction losses (long coil length).

### Validation of the mathematical modeling for water hammer with pressurized air vessel for pipe coil length L = 15 m and orifice diameter of 4 mm

The results of the mathematical model, for the water hammer with the pressurized air vessel for pipe coil length of 15 m and an orifice diameter (the throttling aperture of the air vessel) of 4 mm, have been validated with the obtained experimental results from the laboratory test rig. The validation of this mathematical model has been performed for the transient water hammer head (Fig. 6a), the transient air head (Fig. 6b) and transient water level (Fig. 6c). The maximum experimental and simulated water hammer heads were 37.76 m and 36.1 m, respectively, showing a maximum deviation of 4.39% (Fig. 6a). In addition, the maximum deviation between the experimental and simulated air head and the water level are 9.5% (Fig. 6b) and 3.94% (Fig. 6c), respectively. The obtained mathematical model results show a good agreement with the experimental results, as shown in Fig. 6a–c.

Due to the good agreement between the experimental and mathematical model results, and as the study of the effect of the orifice diameter, vessel volume and water fraction volume ratio require many experiments, therefore, the mathematical model will be used to examine the effect of these parameters.

### Effect of the orifice diameter on water hammer head, H_P_

**Fig. 7:**
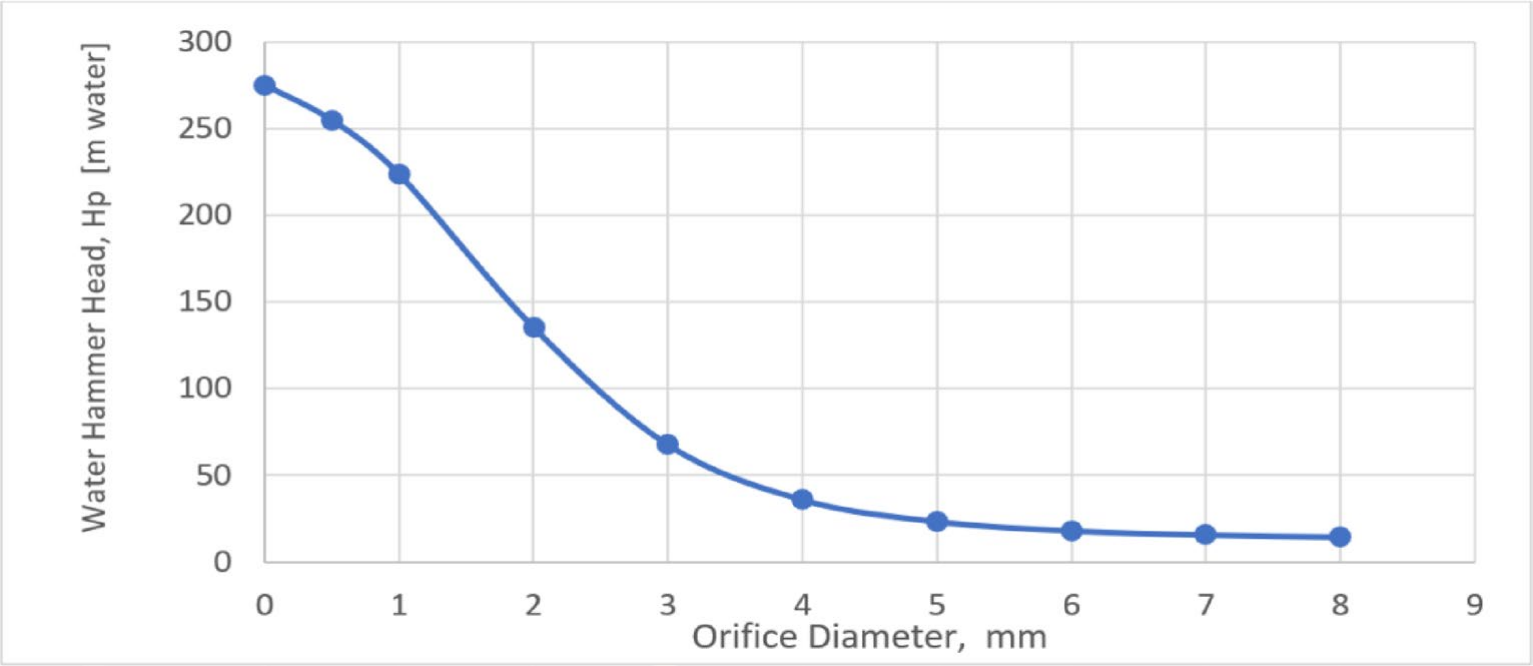
Effect of the Orifice Diameter on the maximum water hammer head.

The orifice diameter (the throttling aperture of the air vessel) plays a major role in suppressing the maximum water hammer head and it should be carefully selected in the piping system. The orifice diameter controls the throttling aperture of the air vessel. Increasing the orifice diameter significantly decreases the water hammer head (HP), as shown in Fig. 7. The water hammer head drops from 275.1 m (without an orifice) to 14.6 m for an 8 mm orifice diameter. The rate of this decrease is particularly high for orifice diameters smaller than 5 mm, where a 91.46% reduction in water hammer head is achieved. Beyond a 5 mm diameter, the effect of the orifice on the water hammer head becomes negligible. In other words, the orifice diameter should be at least 30% of the main pipe’s diameter to be effective.

The exponential reduction in water hammer head with increasing orifice diameter can be attributed to the nonlinear dissipation of surge energy through the orifice opening. At small diameters, the restricted flow capacity allows only limited energy release, resulting in a high-pressure rise. As the orifice diameter increases, the available flow area expands quadratically, permitting a disproportionately larger discharge and consequently a sharp decrease in surge pressure. Beyond a certain size, however, the orifice provides sufficient capacity for energy dissipation, and additional increases yield only marginal reductions in water hammer head. This behavior results in the observed exponential decay, where the pressure head decreases rapidly at smaller diameters and asymptotically approaches a minimum value determined by system inertia and hydraulic losses.

**Fig. 8:**
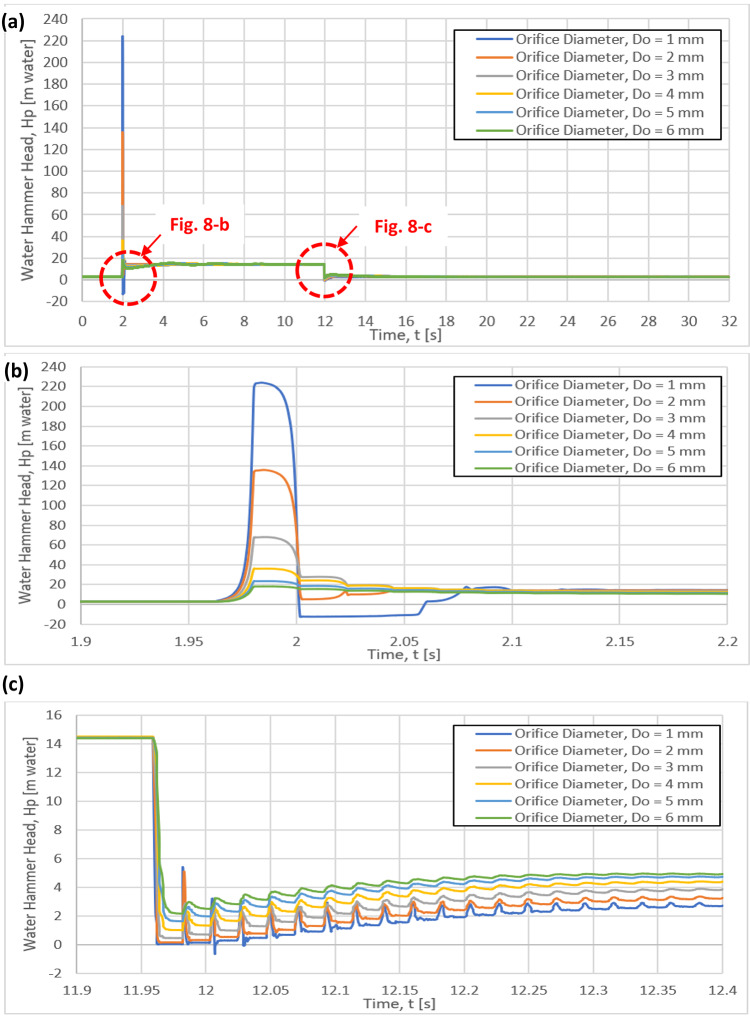
Variation of the Instantaneous water hammer head with the time for different orifice diameters: (**a**) One complete cycle, (**b**) between time 1.9 s to 2.2 s, (**c**) between time 11.9 s to 12.4 s.

The time histories of the water hammer head for six different orifice diameters of 1, 2, 3, 4, 5 and 6 mm are presented in Fig. 8. It can be clearly seen that the six curves of the water hammer head have the same pattern (Fig. 8a), but different values of water hammer heads (Fig. 8b, c). Increasing the orifice diameter from 1 to 6 mm results in a decrease in the maximum water hammer head from 223 m water to 18 m water as shown in Fig. 8b. The steady state water pressure head for the orifice diameter of 6 mm is larger than that of 1 mm, as presented in Fig. 8c.

### Effect of the orifice diameter on vessel air head, H_A_

**Fig. 9:**
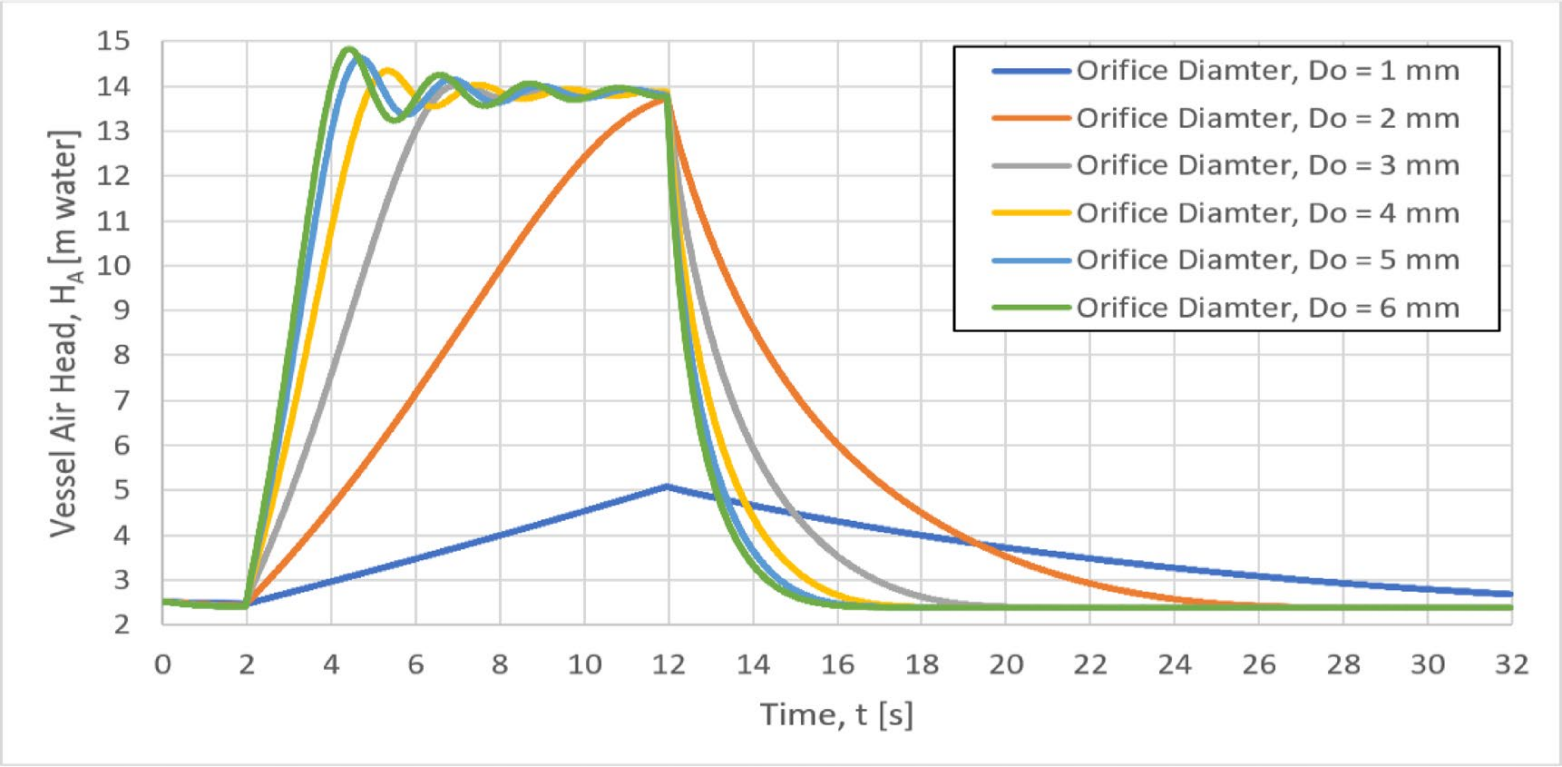
Variation of the air head with the time for different orifice diameters.

The Variation of the vessel air head with the time for six different orifice diameters (the throttling aperture of the air vessel) of 1, 2, 3, 4, 5 and 6 mm is shown in Fig. 9. The slope of the vessel air head, (dH_A_/dt) (time rate of the pressure rise) increases with increasing the orifice diameter from 1 to 5 mm and this slope will remain unchanged approximately for orifice diameter larger than 5 mm. The time rate of pressure rise affects the pipe stresses, therefore using smaller values of the orifice diameter will be better than the larger values as the corresponding pipe stresses will be lower, but the corresponding maximum water hammer head will be larger. Accordingly, the orifice diameter should be carefully selected to have a compromise between the pipe stresses and the water hammer head.

### Effect of orifice diameter on the vessel water level, H_W_

**Fig. 10:**
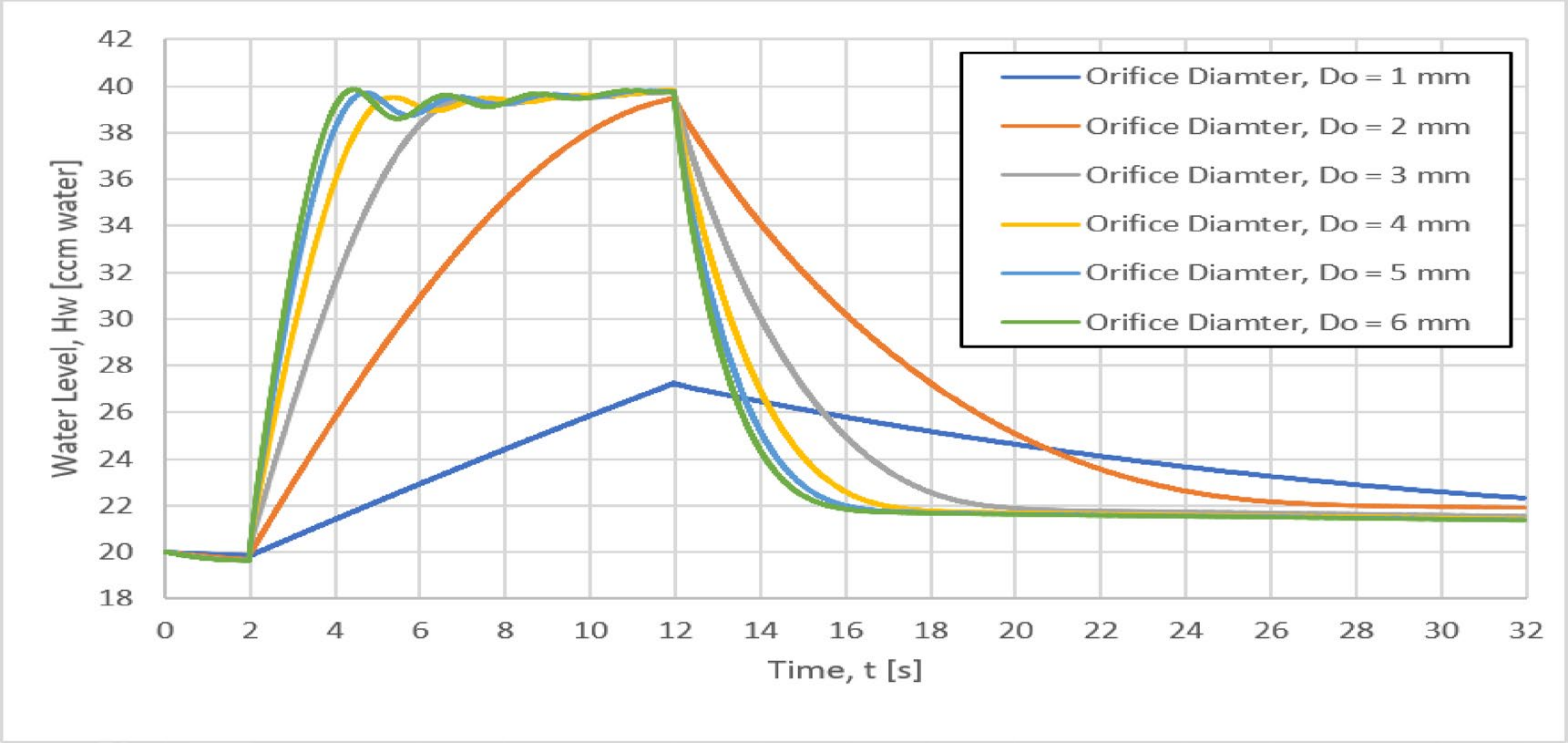
Variation of the Instantaneous water level in the vessel with the time for different orifice diameters.

The water level rise in the vessel determines the required length of the pressurized air vessel as Increasing the water level rise in the vessel will require a longer vessel. The variation of the water level in the pressurized air vessel with the time for different six orifice diameters of 1, 2, 3, 4, 5 and 6 mm is shown in Fig. 10. For the smaller orifice diameter, the water level takes longer time than that of the larger orifice diameter. Consequently, the time rate of the water level rise in the vessel (the slope dH_W_/dt) is increased by increasing the orifice diameter from 1 to 5 mm and this slope will remain unchanged approximately for orifice diameter larger than 5 mm. Moreover, the water level rise is 20 cm (from 20 cm to approximately 40 cm) is almost the same for orifice diameters between 2 and 6 mm. However, the water level rise is smaller (7.1 cm) for the 1 mm orifice diameter, so a shorter vessel length would be required.

### Effect of orifice diameter on orifice discharge, Q_Orifice_

**Fig. 11:**
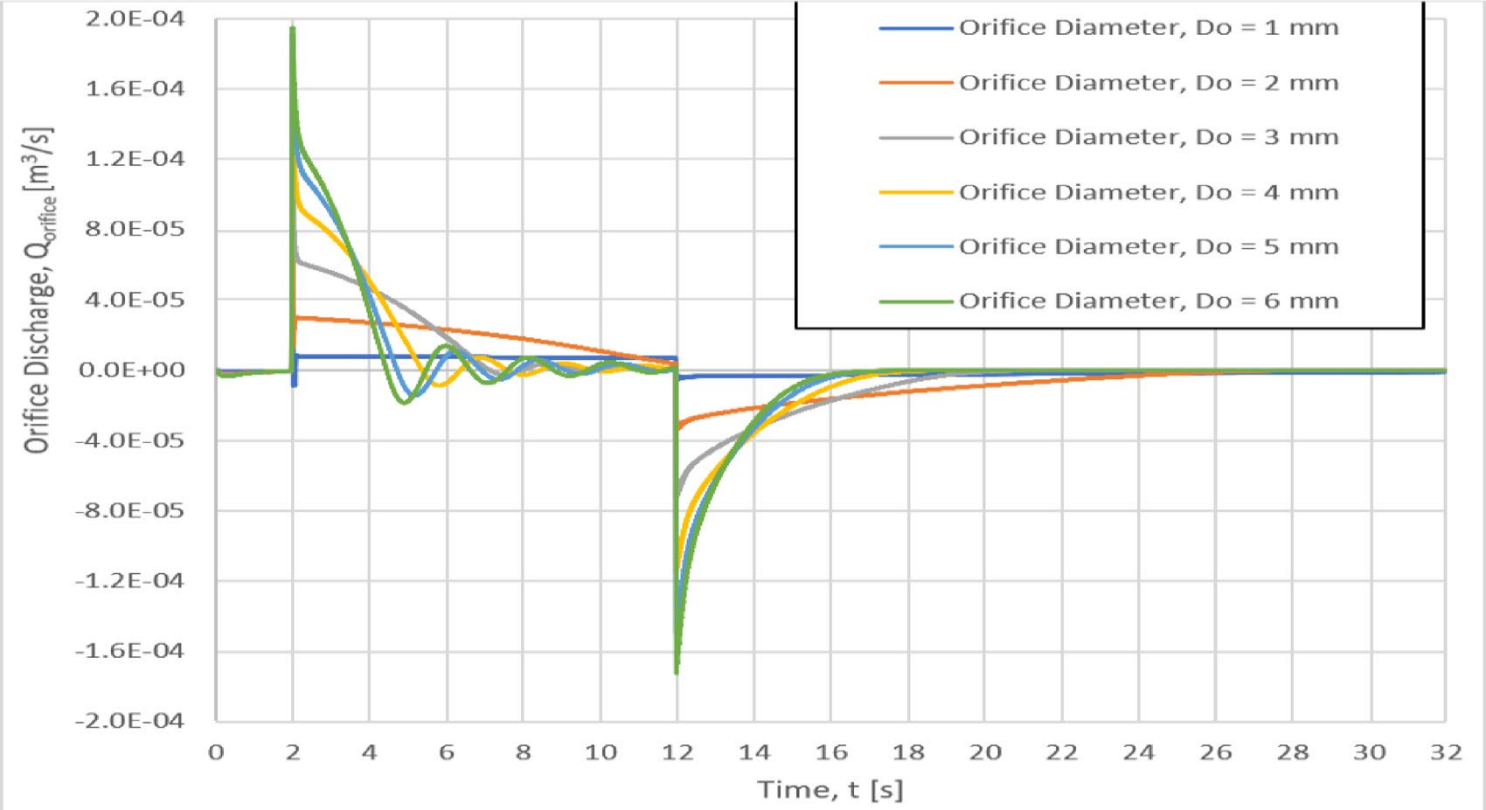
Effect of the orifice diameter on the orifice discharge.

The orifice diameter affects the water discharge through the orifice as shown in Fig. 11 for six different orifice diameters of 1, 2, 3, 4, 5 and 6 mm. It can be seen that the magnitude of the water discharge through the orifice is increased with increasing the orifice diameter accompanied with a flow oscillation and larger discharge slop with the time for orifice diameter lager than 4 mm, as shown in Fig. 11.

### Effect of orifice diameter on the vent mass flow rate, M_O_

**Fig. 12:**
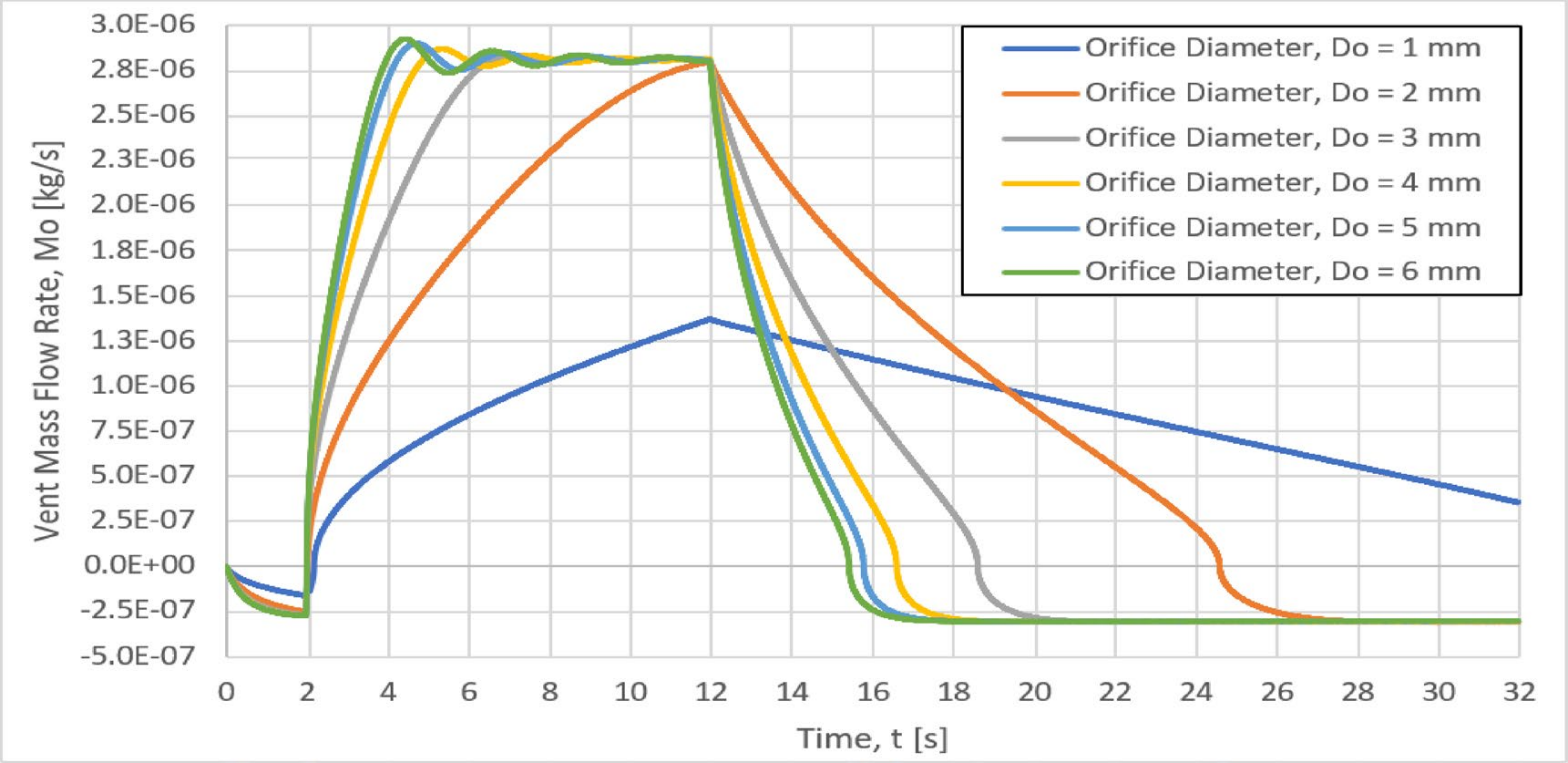
Effect of the orifice diameter on the Instantaneous vent mass flow rate.

The variation of the vent mass flow rate, M_o_ with the time for different six different orifice diameters of 1, 2, 3, 4, 5 and 6 mm, is shown in Fig. 12. Increasing the orifice diameter from 1 to 5 mm, the time rate of the vent mass flow rate (the slop dM_o_/dt) will consequently increase. But, for orifice diameter larger than 5 mm, the slop dM_o_/dt remains approximately unchanged, as shown in Fig. 12. In addition, for orifice diameters larger than 1 mm, the maximum values of the vent mass flow rate are almost the same (2.81E-06 kg/s) as shown in Fig. 12.

### Effect of the vessel diameter, D_v_ on the water hammer head, H_P_, the vessel air head, H_A_, the water level, H_W_ for constant vessel volume and orifice diameter D_o_ = 6 mm

**Fig. 13:**
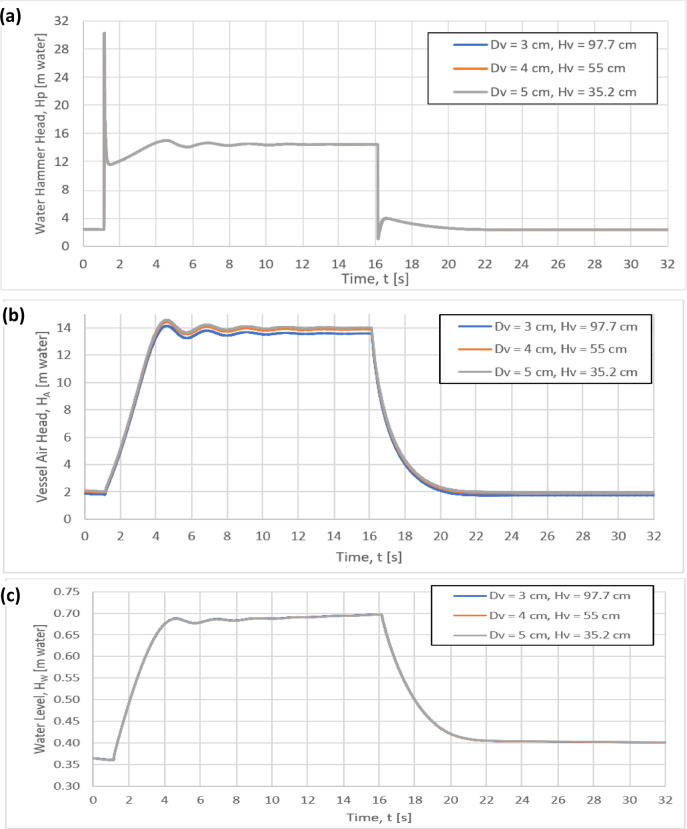
Effect of the vessel diameter, D_v_ for constant vessel volume and orifice diameter D_o_ = 6 mm on: (**a**) the Instantaneous water hammer head, (**b**) the Instantaneous vessel air head, H_A_ and (**c**) the Instantaneous water level, H_W_.

The selection of the vessel diameter D_v_, is another important design parameter that affects the water hammer head H_P_, the vessel air head H_A_ and the water level inside the vessel H_W_. The effect of the vessel diameter D_v_ for constant vessel volume of 691.15 cm^3^ and an orifice diameter of 6 mm on the previously examined parameters are shown in Fig. 13. The vessel diameter does not affect both the water hammer head and the water level for constant vessel volume, as shown in Fig. 13a, c. There are little changes (2.85%) in the maximum vessel air head from 13.6 m water to 14 m water for vessel diameters of 3 cm and 5 cm, respectively, as shown in Fig. 13b.

### Effect of vessel diameter, D_v_ on the water hammer head, H_P_, the vessel air head, H_A_, the water level, H_W_ for variable vessel volume and orifice diameter D_o_ = 6 mm

**Fig. 14:**
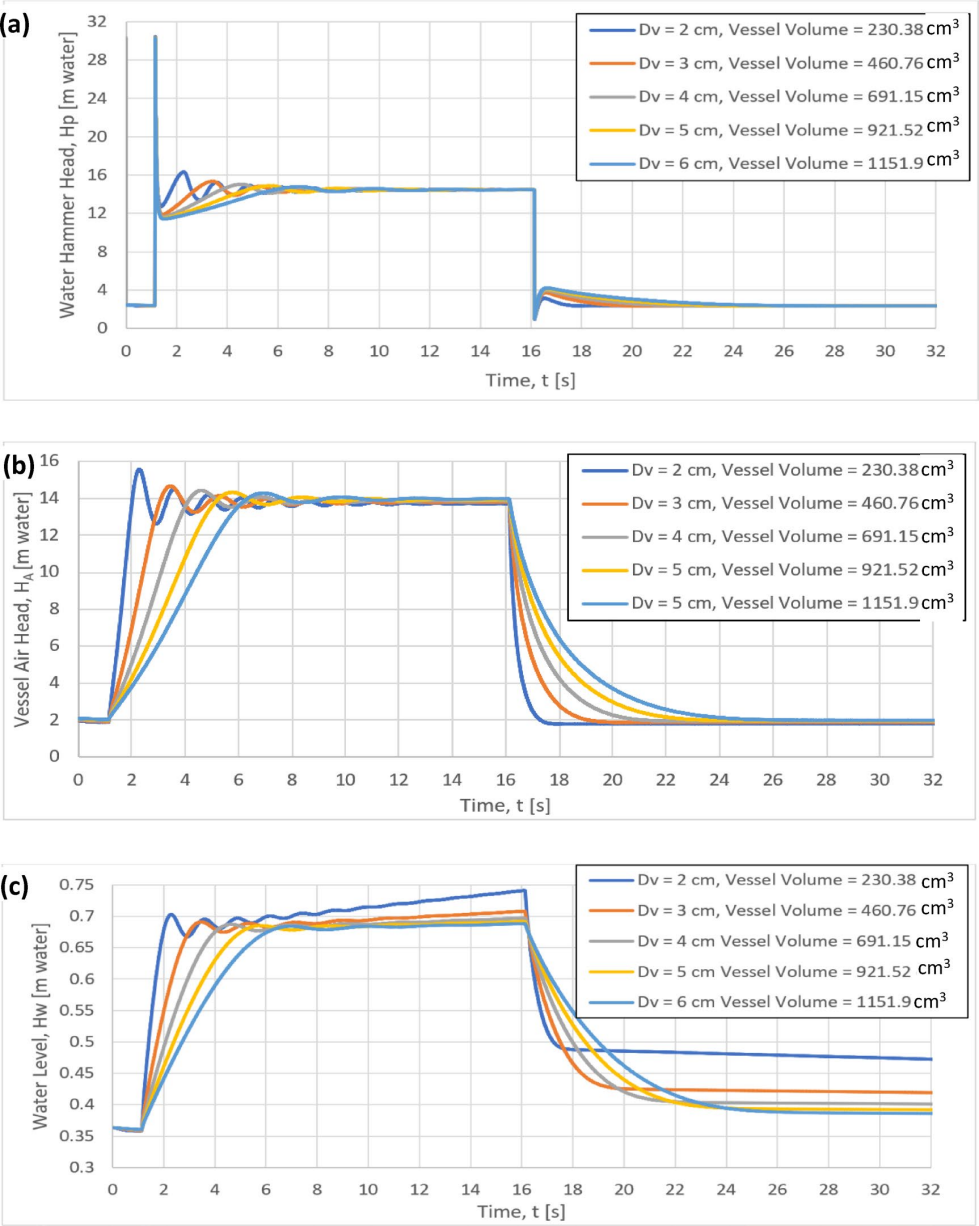
Effect of the vessel diameter, D_v_ for variable vessel volume and orifice diameter D_o_ = 6 mm on: (**a**) the Instantaneous water hammer head, (**b**) the Instantaneous vessel air head, H_A_ and (**c**) the Instantaneous water level, H_W_.

To examine the effect of the vessel diameter D_v_ for variable vessel volume on the water hammer head H_P_, the vessel air head H_A_ and the water level inside the vessel H_W_, five different values of the vessel volume have been considered, 230.38, 460.76, 691.15, 921.52 and 1151.9 cm^3^, for constant orifice diameter of 6 mm, as shown in Fig. 14a–c. The maximum value of the water hammer head is almost the same (30.2 m water) for all the examined cases as shown in Fig. 14a. In addition, increasing the vessel diameter from 2 to 6 cm, the slope (dH_A_/dt) decreases after the maximum value of the water hammer head until reaching the steady state value of 14.5 m water as shown in Fig. 14a. Furthermore, both the time rates of change of vessel air head (dH_A_/dt) and the water level (dH_W_/dt) are decreasing with increasing the vessel diameter for the variable vessel volume, as shown in Fig. 14b, c respectively. It can be concluded that the most effective parameter on the water hammer head, vessel air head and the water level inside the vessel is the orifice diameter.

### Effect of water volume fraction ratios (WVFR) on the water hammer head for different orifice diameters

**Fig. 15:**
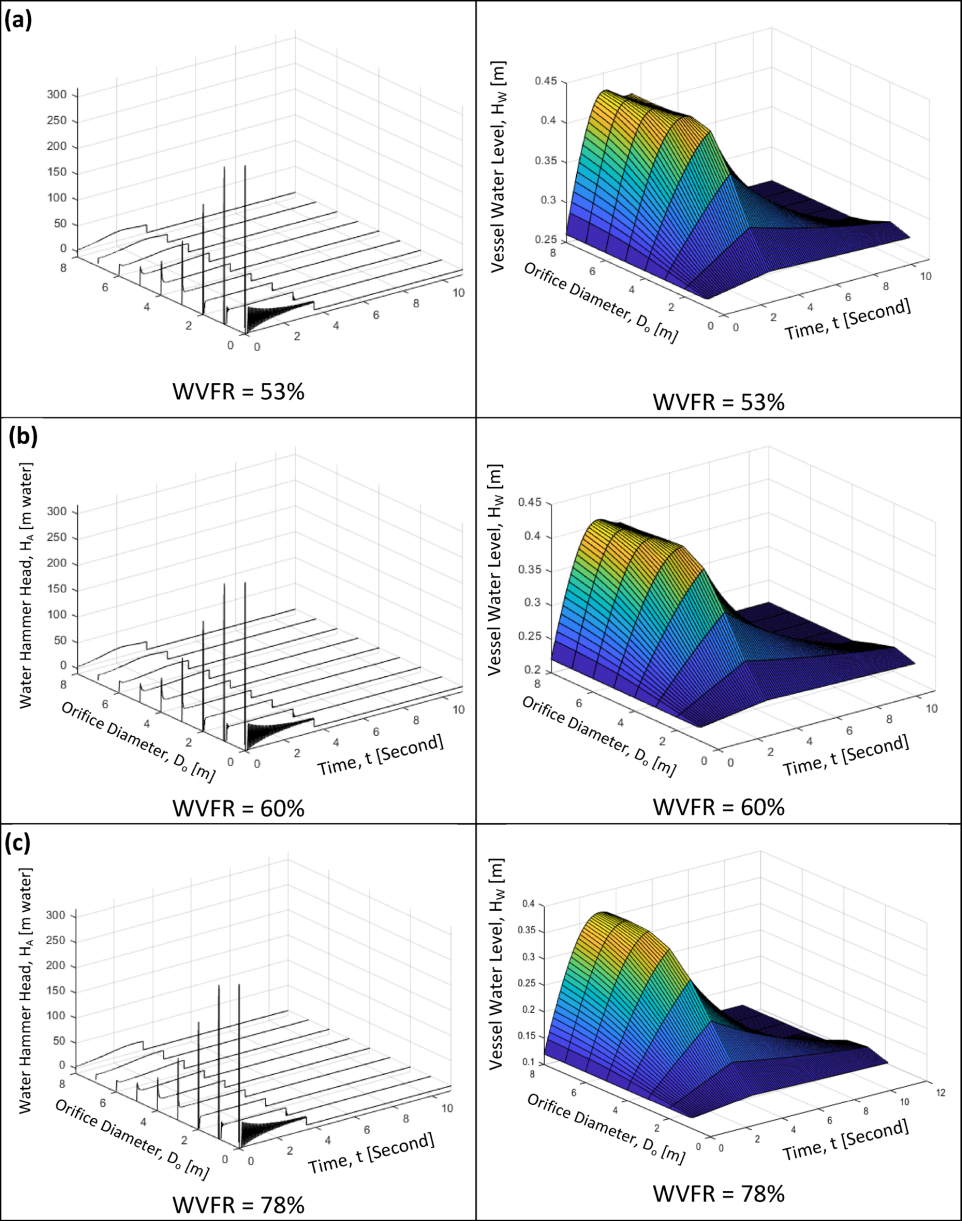
The effect of the orifice diameter on the instantaneous water hammer head for different orifice diameters, D_o_ (1 mm: 8 mm) and three different water volume fraction ratios (WVFR) obtained by the mathematical model: (**a**) WVFR = 53%, (**b**) WVFR = 60% and (**c**) WVFR = 78%

The water volume fraction ratios (WVFR) is defined as the water volume inside the pressurized air vessel to the total vessel volume and it expresses the initial trapped air volume inside the pressurized air vessel. It is an important design parameter that affects the values of the maximum water hammer head. Three different values of the water volume fraction ratios (WVFR) have been examined in the current study, which are 53%, 60% and 78%, for orifice dimeters range from 1 to 8 mm calculated by the mathematical model as shown in Fig. 15. For the water volume fraction ratio (WVFR) of 53%, when increasing the orifice diameter from 1 to 5 mm, the maximum water level inside the vessel will increase from 0.26 m to almost constant value of 0.435 m. In addition, there is no variation in the maximum water level with increasing the orifice diameter above 5 mm, as presented in Table 1. The same behaviour can be seen for the water volume fraction ratio (WVFR) of 60%, where the initial and the maximum water levels are 0.22 m and 0.41 m, respectively. A different behaviour can be seen for the water volume fraction ratio (WVFR) of 78%, that increasing the orifice diameter from 1 to 6 mm, the maximum water level is increased from the initial value of 0.115 m to an almost fixed the maximum value of 0.365 m as shown in Table 1. It can be concluded that increasing the water volume fraction ratio (WVFR), the water level is decreased for the same orifice diameter.

**Table 1:** Comparison of related studies on pressurized air vessels for water hammer mitigation.

Study	Methodology	Parameters investigated	Key findings	Limitations	Contribution of current study
Sattar et al.^17^	Analytical & experimental	Initial air volume (20–75% of vessel)	Recommended range for air volume fraction to suppress surge	Did not study throttling/orifice effect	Current study provides quantitative optimization of orifice diameter and WVFR with validation
Kim et al.^16^	Experimental	Air chamber effect in pump-rising pipeline	Showed chamber reduces surge	Limited to specific pump system	Current study generalizes vessel design using model + experiments
Sun et al.^14^	Numerical (SQP optimization)	Vessel sizing in long-distance supply	Proposed optimization method for sizing	No validation with experiments	Current study combines modeling with experimental validation
Miao et al. ^15^	Simulation	Combined vessel and valve	Hybrid control reduces surge pressure	Focused on long-distance supply only	Current study isolates orifice, vessel volume, WVFR impacts
Izquierdo et al. ^11^	Neural network modeling	Vessel design prediction	Showed AI can approximate vessel design	Lacked experimental support	Current study validates model experimentally and quantifies effects
Martino & Fontana ^32^	Simplified analytical approach	Throttled chambers	Provided sizing charts	Not verified experimentally	Current study experimentally validates throttling effect
Besharat et al.^35^	Experimental	Vessel for energy storage & protection	Demonstrated vessel can mitigate surge	No optimization of parameters	Current study focuses on optimization of key parameters
Current study	Mathematical model (FORTRAN, MOC) + experimental rig	Orifice diameter, vessel diameter, WVFR	Identified orifice as dominant factor; optimum WVFR ≈ 60%; vessel diameter minor effect	Limited to laboratory scale	Provides validated model + parametric optimization for practical design guidelines

Furthermore, the variation of Instantaneous water hammer head with time for eight different orifice diameters from 1 to 8 mm and three different water volume fraction ratios (WVFR) of 53%, 60% and 78% is presented in Table 1. Increasing the orifice diameters from 1 to 5 mm, the maximum value of the water hammer head in clearly decreased. In addition, the maximum value of the water hammer head is slightly decreased with increasing the water volume fraction ratio (WVFR) as shown in Fig. 15.

A comparison for past work-related work done and with the current study is clearly presented in Table 1 that highlights what was done in prior work versus the current contribution. This makes the novelty of the current study clearer. Table 1 clearly shows that while past studies addressed vessel sizing, air volume, or system-level optimization, the novelty of the current study is the combined modeling with the experimental validation, with a focus on quantifying the influence of orifice diameter, vessel diameter, and water volume fraction ratio (WVFR)**.**

## Conclusions

The pressurized air vessel demonstrates a good ability to control the upper and lower pressure limits inside the pipeline. In the current study, the effect of the orifice diameter, the water volume fraction ratios (WVFR), the vessel diameter for both constant and variable vessel volumes on the performance of pressurized air vessel is modelled mathematically and tested experimentally. From the obtained results, the following points can be concluded, as follows:

1. The developed quasi-one-dimensional mathematical model effectively simulates the performance of a pressurized air vessel behaviour during transient flow conditions. The strong agreement between the mathematical model and the experimental results validates its use as a tool for optimizing air vessel parameters.
2. Throttling the aperture of the pressurized air vessel provides a resistance to the inlet flow which affects the size of the air vessel and makes the trapped air to be compressed gradually. In addition, it reduces the mass oscillation of water, which reduces the size of the air vessel.
3. The orifice diameter is the most influential parameter affecting the water hammer head, vessel air head, and water level, with a trade-off required between water hammer head and pipe stresses. Optimal sizing of the orifice is crucial. The orifice diameter should be at least 30% of the main pipe’s diameter to be effective. Iit is a compromise between reducing water hammer head and maintaining acceptable pressure rise rates.
4. The magnitude of the water discharge through the orifice is increased with increasing the orifice diameter accompanied by a flow oscillation and larger discharge slop with the time for orifice diameters larger than 4 mm. Moreover, the time rate of the air pressure rise inside the pressurized air vessel (dH_A_/dt) is increased with increasing the orifice diameter from 1 to 5 mm and this rate remains unchanged approximately for orifice diameter larger than 5 mm.
5. For the smaller orifice diameters, the water level takes longer time than that of the larger orifice diameter. Consequently, the time rate of the water level rise in the vessel (dH_W_/dt) is increased with increasing the orifice diameter from 1 to 5 mm and this slope will remain unchanged approximately for orifice diameter larger than 5 mm. on other hand, when increasing the orifice diameter from 1 to 5 mm, the time rate of the vent mass flow rate (dM_o_/dt) will consequently increase. But, for orifice diameters larger than 5 mm, the rate (dM_o_/dt) remains approximately unchanged.
6. For constant vessel volume, the vessel diameter does not affect both the water hammer head and the water level. While, for a variable vessel volume, both the time rates of change of vessel air head (dH_A_/dt) and the water level (dH_W_/dt) inside the pressurized air vessel are decreasing with increasing the vessel diameter.
7. The water volume fraction ratio (WVFR) is another critical design parameter which expresses the initial volume of the trapped air which is limited between lower and upper values. The optimal WVFR is approximately 60%. While the WVFR can range effectively from 53 to 78% for effective damping, values above 60% show no further benefit in reducing water hammer pressure. Increasing the water volume fraction ratio (WVFR), the water level is decreased for the same orifice diameter, accordingly, a shorter vessel length is required. The maximum value of the water hammer head is slightly decreased with increasing the water volume fraction ratio (WVFR). Furthermore, Increasing the water volume fraction ratio (WVFR) decreases the sudden pressure build-up rise due to water hammer, if the air does not enter the pipeline during the suction period.

Future research should expand upon this work in several directions. Advanced computational fluid dynamics (CFD) simulations, coupled with the method of characteristics, could provide deeper insight into the multiphase interactions between water and compressed air within the vessel. Further investigations should also explore the influence of unsteady friction models**,** pump power failure scenarios, and variable flow rates, which were not fully addressed in this study. Additionally, the role of the air polytropic index and heat transfer effects on vessel performance requires systematic evaluation. On the experimental side, testing under field-scale conditions with varying pipeline materials and geometries would enhance the generalizability of the findings. Finally, multi-objective optimization approaches, incorporating both hydraulic safety and economic cost, could be developed to guide the practical design of surge protection systems in complex water transmission networks.

## Data Availability

The corresponding author can provide the datasets created and/or analysed during the current work upon reasonable request.

## List of symbols

$A_{1}$: Pipe area of upstream air vessel (m^2^)
$A_{2}$: Pipe area of downstream air vessel (m^2^)
$A_{vessel}$: Area of the air vessel (m^2^)
$A_{orifice}$: Area of the orifice (m^2^)
$$a$$: Sound speed (m/s)
C_1_, C_2_, C_3_: Coefficients of pump equation
$$C_{d}$$: Discharge coefficient
D: Diameter of the pipe (mm)
E: Young’s modulus
e: Thickness of the pipe (mm)
$$f$$: Friction coefficient
g: Gravitational acceleration (m/s^2^)
H: Pressure head (m)
K: Bulk modulus (Pa)
k: Iteration level
L: Length of the pipe (m)
N: Grid mesh points number
$p_{air}$: Absolute pressure of air (Pa)
$$p$$: Pressure (Pa)
$Q$: Water flow rate (m^3^/s)
S: Sample
s: Second
$T_{c}$: Closure time of valve (s)
t: Time of wave travel (s)
$$\Delta t$$: Time step (s)
$V_{o}$: Velocity of the flow (m/s)
$$V$$: Velocity (m/s)
$\forall_{air}$: Volume of trapped air inside pressurized air vessel (m^3^)
$\forall_{water}$: Volume of water inside pressurized air vessel (m^3^)
$\forall_{vessel}$: Volume of pressurized air vessel (m^3^)
$x$: Distance along pipe (m)
$\Delta x$: Mesh size in x-direction (m)
$$z$$: Elevation (m)
$Z_{w}$: Height of water column inside air vessel (cm)

## Abbreviations

C^+^: Positive characteristics equation
C^-^: Negative characteristics equation
DAQ: Data acquisition synchronization system
GA: Genetic algorithm
L: Left point
NC: Normally closed
ORD: Orifice diameter
ODEs: Ordinary differential equations
PDEs: Partial differential equations
p: Point
R: Right point
RCV: Rapid closing valve
WVFR: Water volume fraction ratio $(WVFR) = \forall_{water} /\forall_{vessel}$
WTC: Water transmission system
WH: Water hammer
w: Water
MOC: Method of characteristics

## Greek symbols

$\rho$: Density of water (kg/m^3^)
$\beta$: Diameter ratio (orifice diameter/pipe diameter)
$$\gamma$$: Polytropic index
